# Evaluation of Focus Measures for Hyperspectral Imaging Microscopy Using Principal Component Analysis

**DOI:** 10.3390/jimaging10100240

**Published:** 2024-09-26

**Authors:** Humbat Nasibov

**Affiliations:** National Metrology Institute, The Scientific and Technological Research Council of Türkiye (TÜBİTAK-UME, Ulusal Metroloji Enstitüsü), Kocaeli 41470, Türkiye; humbet.nasibli@tubitak.gov.tr or humbat.nasibov@gmail.com

**Keywords:** autofocus, focus measures, focus criteria, hyperspectral microscope, principal component analysis, PCA, soil reflectance spectrum

## Abstract

An automatic focusing system is a crucial component of automated microscopes, adjusting the lens-to-object distance to find the optimal focus by maximizing the focus measure (FM) value. This study develops reliable autofocus methods for hyperspectral imaging microscope systems, essential for extracting accurate chemical and spatial information from hyperspectral datacubes. Since FMs are domain- and application-specific, commonly, their performance is evaluated using verified focus positions. For example, in optical microscopy, the sharpness/contrast of visual peculiarities of a sample under testing typically guides as an anchor to determine the best focus position, but this approach is challenging in hyperspectral imaging systems (HSISs), where instant two-dimensional hyperspectral images do not always possess human-comprehensible visual information. To address this, a principal component analysis (PCA) was used to define the optimal (“ideal”) optical focus position in HSIS, providing a benchmark for assessing 22 FMs commonly used in other imaging fields. Evaluations utilized hyperspectral images from visible (400–1100 nm) and near-infrared (900–1700 nm) bands across four different HSIS setups with varying magnifications. Results indicate that gradient-based FMs are the fastest and most reliable operators in this context.

## 1. Introduction

A hyperspectral imaging technology that was initially developed for remote sensing and military applications has thoroughly spread over vast application areas and disciplines. For instance, it has been equally successful when applied to distinguishing tumors from normal tissue [1,2], as well as in astronomy for investigations into the distribution of mineralogy on a global scale on the Martian surface [3]. This spacious progress became possible due to many mutually supporting factors, such as advances in multivariate data analysis methods and improvements in high-output optics and solid-state imaging sensors.

Extensive research in the field of optical design resulted in imaging spectrographs with a significantly improved throughput, which in conjugation with highly sensitive digital cameras conceive a new paradigm in optical microscopy—high-magnification, high-resolution hyperspectral imaging microscopy. Even the first hyperspectral imaging microscopes (HSIMs), which covered only the visual spectral region, demonstrated many advantages over the traditional color imaging microscopes. The advantages were mainly due to the capability of differentiating among similarly perceived objects [4]. Additionally, recent achievements in uncooled high-resolution InGaAs focal plane arrays (FPAs) have allowed for integrating the benefits of vibrational spectroscopy with high-magnification short-wavelength infrared (SWIR) microscopy. As a result, it enables us to sense the spatial distribution of various chemical components, whose spectral signatures originate from molecular overtones and combination vibrations in the SWIR range (700–2500 nm). The ability of HSIMs to recover the chemical constituent maps of investigated samples has significantly extended their application areas and made them an important tool in many fields for the estimation and prediction of the comprising components [5,6,7,8]. More importantly, the integration of chemometrics with the near-infrared (NIR) hyperspectral imaging has transformed HISM from a tool that was used only for the detection and visualization of physical, chemical, and biological quality attributes’ distribution to a measurement instrument that allowed for quantitatively assessing these attributes [9]. Recently, the introduction of deep learning methods in hyperspectral content analyses has significantly increased the ability of hyperspectral imaging to classify heterogeneous samples using their spectrally and spatially resolved content. This transformation has turned HSIM into a major microscopy technique that is widely adopted not only by chemometricians and analysts but also in diverse multidisciplinary fields [10]. An explosive growth of applications is observed in safety and quality control [11] and the inspection of anomalies [12], in food commodities and food adulteration [13,14], and in cultivated land quality [15]. The spectral sensing techniques are commonly used for estimation spectral signatures of various substances including the human coronavirus [16] and land cover [17].

The performance of multivariate statistical algorithms in the extraction of reliable chemical information from hyperspectral images strongly depends on the quality of the band images that constitute analyzed hypercubes. Focusing affects image quality and is one of the fundamental adjustment operations available in optical imaging instruments. A well-focused image has the highest achievable sharpness, has large contrast, and contains a considerable amount of details (within the field of view (FOV)) by means of a wide range of intensity values [18].

The focusing can be performed manually or automatically. The latter process is called autofocusing. Unfortunately, a universal autofocusing algorithm (AFA) does not exist. In-stead, the AFA selection is made for each specific imaging domain. The performance of various AFAs across a range of imaging applications—including optical microscopy [19], automated microscopy for biological samples [19,20,21,22,23], optical systems [24,25,26,27,28], digital holographic microscopy [29], thermal imaging [30,31], and scanning electron microscopy [32]—has been extensively compared and studied. However, to the best of our knowledge, there is no comprehensive study of the performance of AFAs within the hyperspectral imaging domain.

The aim of the current work is twofold. The first objective is to establish a benchmark for the autofocusing of HSIS by evaluating twenty of the most widely used FMs, carefully selected based on their performance across various imaging domains. The other aim is to demonstrate that PCA, employed as a reference point to determine the optimal focus position, outperforms human-comprehensible visual information, while instantaneous two-dimensional hyperspectral images do not always possess such clarity.

The remainder of this paper is organized as follows: Section 2 describes the developed test facility including measurement methods and main metrological characteristics. Thereafter, the results of the experimental measurements are discussed in Section 3. Finally, a discussion and conclusions are presented in Section 4 and Section 5, respectively.

## 2. Materials and Methods

### 2.1. Autofocus Methods

In an image acquisition process, the objective of an AFA is to automatically adjust the lens to the correct position (in focus), ensuring that light from a single point on the object plane converges to a single point on the image plane. Consequently, the resulting image on the focal plane exhibits higher sharpness, with its FM function reaching a global extremum, typically a maximum.

In automated microscopy, a series of *N* images, each corresponding to the same FOV but captured at different focal positions around the focal plane, are acquired. This sequence is obtained by moving the sample along the optical axis of the microscope system, typically using a servo-controlled translation stage. The images in the stack are indexed based on their focal positions along the optical axis. Given that the images encompass the same FOV, the sole distinction lies in their sharpness. According to this setup, the image with the highest sharpness is anticipated to represent the best focus [18]. Thus, for each image in the stack, a scalar value of the FM is computed and stored in a vector. Subsequently, by seeking a global maximum in the vector, the optimal focal position is determined.

**Figure 1:**
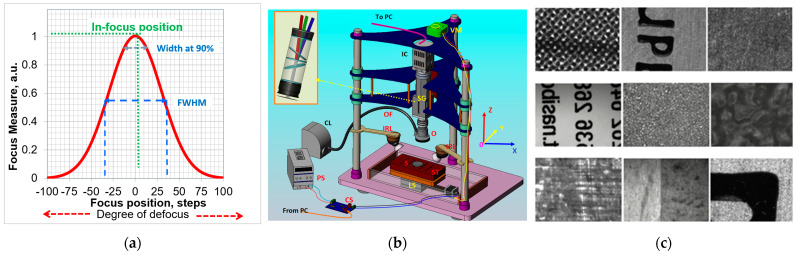
(**a**) A characteristic FM curve; (**b**) a schematic of HSIMs: IC—imaging camera, VM—precise vertical translation stage, SG—spectrograph, OF—optical fiber, O—objective lens, IRL—infrared lamp, CL—collimated light source, S—sample, ST—sample table, LS—translation linear stages, PS—power supply, PC—personal computer; (**c**) in-focus images of the samples used in the experiments.

The primary figure of merit for any FM is its focus curve, which represents the relationship between image sharpness and focal position. Ideally, an effective FM should exhibit a smooth focus curve featuring a single, clearly defined peak corresponding to the precise focus position (Figure 1a). Additionally, the curve should symmetrically and uniformly decrease (or increase) on either side of the focus position. However, in practical scenarios, focus curves may significantly deteriorate and deviate from the ideal expectations. The robustness of the focus curve is not solely contingent on the specific FM employed; rather, factors such as the noise level, image content, and characteristics of the imaging system can contribute to the degradation of the curve.

A brief description of the 22 FMs utilized in this study is listed in the next subsection.

### 2.2. The Focus Measures

In the present study, we evaluate a collection of twenty-two FMs within the context of autofocusing in hyperspectral imaging, which possesses its own peculiarities. Below is the brief description of each FM.

*i. Gradient-based functions.*

The gradient-based focus measures rely on intensity variations among neighboring pixels, exploiting the principle that focused images exhibit higher-intensity differences. These functions calculate the local gradient of each individual image in the stack and then sum them:

$${FM}_{grad}=\sum_{i,j}{\left\|\nabla I\left(i,\left.j\right)\right.\right\|}^{2},if\;\nabla I\left(i,j\right)>\theta$$

where $\left\|\circ \right\|$ denotes the Euclidean norm. To enhance sensitivity in noisy images, an accumulation of gradient values above a threshold value $\left(\theta \right)$ is commonly performed. The most popular gradient-based focus measure introduced by Tenenbaum is considered the benchmark in the field [33,34]. It convolves an image with two two-dimensional filter kernels $\left(O_{p}\right)\;(p=1\;and\;2)$ and then sums the square of the gradient vector components:

$${FM}_{grad}=\sum_{i,j}\left({\left(I*O_{1}\right)}_{i,j}^{2}+{(I*O_{2)}}_{i,j}^{2}\right),$$

where “∗” indicates the two-dimensional discrete convolution operator, and the kernels are defined as

$$O_{1}=\left[\begin{array}{ccc} -1 & 0 & 1\\ -a & 0 & a\\ -1 & 0 & -1 \end{array}\right]\mathrm{and}\;O_{2}=O_{1}^{T}.$$

In this study, we employ two pairs of kernels, where the specific parameter value a determines the FM variant. For a=2, the FM is termed as Tenengrad FM on Sobel operators (referred to as ***TEN1***), and for a=1, it is denoted as Tenengrad FM on Prewitt operators (***TEN2***). Both FMs are based on standard edge detection masks with different kernels. ***TEN2*** is used to detect horizontal and vertical edges, while ***TEN1*** emphasizes central pixels and is less sensitive to noise. 

Additionally, three FMs based on the second derivatives, such as

$${FM}_{grad}=\sum_{i,j}{\left\|\nabla^{2}I(i,j)\right\|}^{2}$$

were included in the scope of the present work.

(i) Energy of Laplacian (***EOL***): This FM retrieves the sharpness value by analyzing high spatial frequencies associated with image borders and is computed by convolving an image with the convolution mask given by
  $$EOL=\sum_{i,j}{\left({\left(I*L_{x}\right)}_{i,j}+{\left(I*L_{y}\right)}_{i,j}\right)}^{2},$$
  where $L_{x}=\left[\begin{array}{ccc} -1 & 2 & -1 \end{array}\right]$ and $L_{y}=L_{x}^{T}$ are the Laplacian operators.
(ii) Sum-Modified Laplacian (SML): Proposed by Nayer and Nakagawa [34], this function serves as an alternative definition of EOL. It is derived from the observation that horizontal and vertical directions can have opposite signs, canceling each other out:
  $$SML=\sum_{i,j}{\left|I*L_{x}\right|}_{i,j}+{\left|I*L_{y}\right|}_{i,j}.$$
(iii) Diagonal Laplacian (***DLF***): This focus measure, proposed by [35], extends the SML with diagonal terms, thus considering variations in both directions, i.e., along the spectral and spatial directions in hyperspectral images. It was subjected to evaluations in this work:
  $$DLF={\left({\left|I*L_{x}\right|}_{i,j}+{\left|I*L_{y}\right|}_{i,j}+{\left|I*L_{d1}\right|}_{i,j}+{\left|I*L_{d2}\right|}_{i,j}\right)}^{2},$$
  where
  $$L_{d1}=\frac{1}{\sqrt{2}}\left[\begin{array}{ccc} 0 & 0 & 1\\ 0 & -2 & 0\\ 1 & 0 & 0 \end{array}\right]\mathrm{and}\;L_{d2}=\frac{1}{\sqrt{2}}\left[\begin{array}{ccc} 1 & 0 & 0\\ 0 & -2 & 0\\ 0 & 0 & 1 \end{array}\right].$$

Next, three representatives of another family of gradient-based FMs, which accumulate image derivatives of the generalized form

$${FM}_{der}=\sum_{i,j}{\left(I\left(i,j\right)-I(j+k,j+l)\right)}^{p},$$

under the condition $\left(I\left(i,j\right)-I(i+k,j+l)\right)>\theta ,$ were included in this work:

(iv) ${FM}_{der}$ with parameters l=0,k=1,p=1, and θ>0 is named the Thresholded Absolute Gradient, and referred to as ABG in this paper. The ***ABG*** is based on summing the first derivative of the image in the horizontal dimension, as a focused image has more gradients than a defocused image.
(v) The case with parameters (l=0,k=1,p=2) is named the Squared Absolute (SAG). The SAG is distinguished from the ABG y summing the square of the first derivative of the image in the horizontal dimension, to increase the contribution of larger gradients.
(vi) The case with parameters (l=0,k=2,p=2) is named the Brenner function (BRE) [32]. This focus measure (FM) is based on the second difference of the image intensity in the horizontal direction, which corresponds to the spatial axis of hyperspectral images. Some works also report applying it in the vertical direction.

The SAG and BRE can be used without applying a threshold; however, in the current work, we applied a threshold value based on the noise level of the images.

Finally, we complete the list of gradient-based FMs with the following two functions:

(vii) Energy of Image Gradient (***EIG***): This measure accumulates the sum of squared directional gradients, given by the following [36]:
  $$EIG=\sum_{i,j}{\left(I_{x}\left(i,j+1\right)+I_{y}\left(i,j\right)\right)}^{2},$$
  where $I_{x}\left(i,j\right)=I\left(i+1,j\right)-I\left(i,j\right)$, and $I_{y}\left(i,j\right)=I\left(i,j+1\right)-I\left(i,j\right).$
(viii) Boddeke’s Algorithm (***BOD***): This function relies on computing a gradient magnitude value using a one-dimensional convolution mask, $B_{x}=\left[-1\;0\;1\right],$ specifically along a single direction [27]. In the evaluations of hyperspectral image stacks, this direction corresponds to the spatial information dimension.
  $$BOD=\sum_{i,j}{\left(\nabla I\left(i,\left.j\right)\right.\right)}^{2},$$
  where $\nabla I\left(i,\left.j\right)\right.=I\left(i+1,\left.j\right)-I\left(i-1,\left.j\right)\right.\right.$.

*ii. Statistically based functions.*

These functions form another family of FMs, involving calculations of image variance, entropy, and correlation. Well-focused images typically exhibit higher variance, information content, and sharper autocorrelation peaks compared to blurred ones [22].

(i) The Normalized Variance of an Image (***NVR***) is based on summing the variance of an image’s gray level with respect to its mean intensity and is defined as
  $$NVR=\frac{1}{\mu WH}\sum_{W}\sum_{H}{\left[I\left(i,j\right)-\mu \right]}^{2},$$
  and here $\mu =\frac{1}{WH}\sum_{W}\sum_{H}I(i,j)\;$ represents the mean intensity; *W* and *H* are the image width and height in pixels.
(ii) The Autocorrelation Function (***ACF***), also known as Vollah’s F4 function, is more robust to image noise and computes the image’s autocorrelation [23]:
  $$ACF=\sum_{W}\sum_{H}I\left(i,j\right)*I\left(i+1,j\right)-\sum_{W}\sum_{H}I\left(i,j\right)*I(i+2,j).$$
(iii) The Standard Deviation-based Autocorrelation Function (Vollah’s F5 function) is utilized, which suppresses high frequencies (***VOL5***) [37]:
  $$VOL5=\sum_{W}\sum_{H}I\left(i,j\right)*I\left(i+1,j\right)-H\cdot W{\cdot \mu }^{2}.$$

Additionally, three image histogram-based functions were included in the evaluation list. These functions offer different insights into the distribution and characteristics of the image histogram, contributing to the assessment of image sharpness and focus.

(iv) Entropy Function (***ENT***): A focused image has higher entropy (i.e., more information) than a defocused image, and therefore, the range of the image histogram can be used as an FM. The *ENT* FM uses the image histogram and is defined as
  $$ENT=-\sum_{l}p_{l}\cdot \log_{2}\left(p_{l}\right),$$
  where $p_{l}=h(l)/\left(H*W\right)p_{l}$ is the probability for each intensity level l in the histogram; h(l) represents the number of pixels with intensity l.
(v) Variance of the Log-histogram (***LOG***): This FM is based on the assumption that high-intensity pixels contribute to the upper part of the histogram and addresses the image’s brightness level through a logarithmic transformation of the histogram [23].
  $$LOG=\sum_{l}{\left(l-E_{log}\left(l\right)\right)}^{2}\log\left(p_{l}\right),$$
  where $E_{log}\left(l\right)=\sum_{l}(l\cdot \log(p_{l})$.
(vi) Weighted Histogram (***WHS***): This FM is based on a weighted image histogram without introducing a constant threshold, taking into account that a focused image has more bright pixels than a defocused image [23]. The values of power and roots are determined empirically. Here, l and h(l) represent the gray level and the number of pixels at each gray level, respectively.
  $$WHS=\sum_{l}(10^{-15}\cdot l^{5}\cdot \sqrt[{5}]{h(l)}),$$

*iii. Frequency domain focus functions.*

They transform the image from the spatial domain to the frequency domain, where more blurred images have fewer high-frequency components. In defocused images, the interaction between adjacent pixels occurs at low frequencies, and as the image becomes more focused, the number of high-frequency components in the frequency domain increases, which serves as the basis for image clarity.

Three widely used representatives of these group FMs were evaluated in the current work [38].

(i) The first is the Fourier transform (***FFT***), which is given by
  $$FT=\sum_{u,v}\sqrt{u^{2}+v^{2}}\cdot \left|G\left(u,v\right)\right|,$$
  and here u and v are the coordinates in the frequency domain, and G is the Fourier transform of the image, where the zero-frequency component is shifted to the center of the Fourier spectrum, i.e., the image array is first zero-padded before performing the FFT. From the real and imaginary parts of each transformed array, the value of the FM is calculated.

The two Discrete Cosine Transform (DCT)-based FMs below require only real number calculations, which improves the speed of the algorithm.

(ii) The second transform-based FM is named as the Discrete Cosine Transform (***DCT***) was calculated using the formula
  $$DCT=C_{u}\cdot C_{v}\cdot \sum_{m,n}I\left(m,n\right)\cdot \cos\left[\frac{\pi \left(2m+1\right)u}{2K}\right]\cdot \cos\left[\frac{\left(2n+1\right)v}{2K}\right],$$
  here $C_{u}=1/\sqrt{K}$ if u=0, or $C_{v}=1/\sqrt{K}$ if v=0, and $C_{u}=C_{v}=\sqrt{2/K}$ in other cases.
(iii) The third function is the Midfrequency-DCT (***MF-DCT***) [29]. This FM is the 8 × 8 modification of DCT, and like DCT is computed for every pixel according to its neighborhood [38]:
  $$MFDCT=\sum_{m,n}I\left(m,n\right)*O_{MDCT},$$
  where
  $$O_{MDCT}=\left[\begin{array}{c} \begin{array}{c} 1\\ 1 \end{array}\\ -1\\ -1 \end{array}\begin{array}{c} \;\begin{array}{c} 1\\ 1 \end{array}\\ -1\\ -1 \end{array}\begin{array}{c} \begin{array}{c} -1\\ -1 \end{array}\\ \;1\\ \;1 \end{array}\begin{array}{c} \begin{array}{c} -1\\ -1 \end{array}\\ \;1\\ \;1 \end{array}\right]$$

Finally, the next three wavelet-based FMs were directly applied as described in [20]. Briefly, the image is decomposed into four sub-images using a discrete wavelet transform. Then, these FMs’ functions utilize sub-images (ll,lh,hl,hh) of an image subjected to the wavelet filter Daubechies-06 of both types: high-pass (h) and low-pass (l) types:

(iv) **(*WL1*)** Wavelet Algorithm: This FM sums the absolute values in (hl,lh,hh) sub-images:
  $$WL1=\sum_{W}\sum_{H}\left|W_{hl}(i,j)\right|+\left|W_{lh}(i,j)\right|+\left|W_{hh}(i,j)\right|;$$
(v) **(*WL2*)** Wavelet Algorithm: This FM uses the variance of wavelet coefficients and sums them in (hl,lh,hh) sub-images. Here, the mean values μ in each region (hl,lh,hh) are computed from absolute values.
  $$\;WL2=\frac{1}{H\cdot W}\sum_{W}\sum_{H}{\left(\left|W_{hl}(i,j)\right|-\mu_{hl}\right)}^{2}+{\left(\left|W_{lh}(i,j)\right|-\mu_{lh}\right)}^{2}+{\left(\left|W_{hh}(i,j)\right|-\mu_{hh}\right)}^{2};$$
(vi) **(*WL3*)** Wavelet Algorithm: The difference between ***WL2*** and ***WL3*** is that the mean values μ are computed without absolute values:
  $$WL3=\frac{1}{H\cdot W}\sum_{W}\sum_{H}{\left(W_{hl}\left(i,j\right)-\mu_{hl}\right)}^{2}+{\left(W_{lh}\left(i,j\right)-\mu_{lh}\right)}^{2}+{\left(W_{hh}\left(i,j\right)-\mu_{hh}\right)}^{2}.$$

### 2.3. PCA

PCA is a strong mathematical tool that is commonly used to transform a multivariate dataset with intercorrelated variables into a reduced-dimensionality set of uncorrelated variables, known as principal components. These principal components are derived through linear combinations of the original variables. The total variance of the principal components is equivalent to the total variance of the original variables, where each component is also associated with a rank indicating the percentage of the data variance it possesses. Depending on the application, various strategies can be followed to select the number of the components [39,40,41,42,43,44].

In this work, we applied PCA to both out-of-focus and focused images, and identified a relationship between the ranking of principal components and image sharpness. The concept of PCA can be found in various sources, such as [39,42]. Here, we briefly describe its implementation within the MATLAB environment [45], which was applied in the current work. Initially, for each image $I_{s}$(s denotes the step number within a single autofocus search cycle), a zero-mean image $I_{m}$ is calculated as $I_{m}={(I}_{s}-\mu )/\sigma$, where μ and σ images represent the mean and standard deviation, respectively. Then, the covariance matrix is computed as $C=(1/WH)I_{m}^{T}I_{m}$, where $I_{m}^{T}$ is the transpose of $I_{m}$, and *W* and *H* are the image dimensions. Following this, the eigenvalues Λ (i.e., principal components) and corresponding eigenvectors V of the correlation matrix C are calculated using the equation CV−ΛV=0, to extract the principal components. Once the images are projected into the PCA domain, the principal components are sorted in decreasing order, with the first principal component corresponding to the largest variance. Since out-of-focus images are typically blurred and exhibit lower variance (i.e., carry less signal) compared to focused images, it is expected that images captured closer to the focal plane will have a higher signal-to-noise ratio, thus accumulating more variance in fewer principal components. We exploit this PCA behavior and calculate a focus curve, where the y axis corresponds to the number of principal components that are required to preserve a certain fixed amount (e.g., 98–99%) of data variance and the × axis, as per usual, corresponds to the focal position. We have observed that the global minima of such a curve correspond well to the best focus position.

### 2.4. Ranking Criteria

The criteria for assessing the performance of the FM may vary depending on imaging systems and specific applications. Various criteria are available for ranking the FMs [20,28,32,46], etc. However, two criteria, namely accuracy and unimodality, are the most important and common for all types of imaging systems.

**Accuracy (**$d_{acc}$**)**: This measure is used to assess how the estimated best focus position, obtained through FMs, deviates from the optical (“ideal”) focus position. Typically, in the literature, the actual focus position is determined by laboratory staff or proficient microscope technicians who visually search for the best focus position, characterized by the image with the highest contrast, sharpest edges, and highest intensity values.

However, in hyperspectral imaging, accurately determining the focus position visually is more challenging compared to conventional imaging systems. Therefore, in this study, the “ideal” focus position was determined using PCA. The focus accuracy is evaluated by the in-focus position error, defined as the absolute difference between the peak positions determined by the PCA analysis and those provided by the given FM. The optimal value is 0.

*Unimodality* ($d_{uni}$): An ideal focus curve should display a single prominent peak corresponding to the optimal focal position. However, in practical scenarios, focus curves may contain several local maxima or minima, which can hinder the accurate determination of the in-focus position. The presence of false extremes is greatly influenced by image noise. The optimal value is 0.

Furthermore, to assess the performance of focus measures in the context of hyperspectral imaging, we have employed three additional criteria.

*Width at 50% of Maximum* ($d_{50}$): This measure, also referred to as Full Width at Half Maximum (FWHM), is utilized to characterize the convergence of the focus curve and the sharpness of its peak. It is defined as the curve width at half of its maximum height.

*Width at 90% of Maximum* ($d_{90}$): In the dynamic line scanning mode of hyperspectral imagers, an autofocus measure should demonstrate sensitivity to changes in the distance between the imager and the sample surface. This sensitivity is vital for UAV-based hyperspectral imaging, where the distance fluctuates due to the topography of the land surface and environmental factors such as wind. Therefore, we introduce the width at 90% of the maximum to improve the performance of the FM in the scanning mode. A smaller value of this measure enables an easier detection of defocusing and speeds up the attainment of the global extremum.

*Smoothness* ($d_{smo}$): Searching for the focus position along a smooth curve is relatively easier compared to a jagged one. While numerous smoothness indices exist [47], we opt for the sum of the absolute values of the first derivative of the curve. A lower smoothness index indicates a smoother curve. Throughout the assessments, we employ a normalized smoothness index.

### 2.5. HSIMs

Throughout the course of the current work, 4 (four) distinct experimental setups (Table 1) employing hyperspectral imaging have been designed and implemented. All of these setups have incorporated a transmission-type pushbroom (line scanning) spectrometer from Specim (Finland), featuring a dispersive element arranged in a prism–grating–prism optical configuration (Figure 1b, inset). Two variations of spectrometers were utilized: one operating in the visible-near-infrared (Vis-NIR) spectral region from 400 nm to 1000 nm and the other in the NIR spectral region from 900 nm to 1700 nm.

Two HSIS, operating in the Vis-NIR and NIR spectral bands, utilize high-magnification microscope objectives from Hirox, Japan (OL-700II and OL-140I, respectively), along with a variable (1×–10×) zoom lens. These configurations are referred to as HSIM1 and HSIM2 throughout this paper. The HSIM setups are primarily used in studies of soil chemical components. A custom-built two-axis computer-controlled translation platform was employed to scan the sample with precise and adjustable scanning speed. Additionally, a step motor-controlled translation stage with a minimum step size of 0.6 μm was utilized to adjust the microscope–sample distance, thereby enabling focal position adjustments (Figure 1b). In the initial setup, illumination was achieved using a current-stabilized tungsten–halogen lamp (up to 250 W), which was focused onto the coaxial entrance of the microscope, ensuring an “ideal” illumination and diffuse detection configuration as described in [8]. To mitigate second-order spectra, a half-colored long-pass cut-off filter was positioned at the output of the spectrometer.

**Figure 2:**
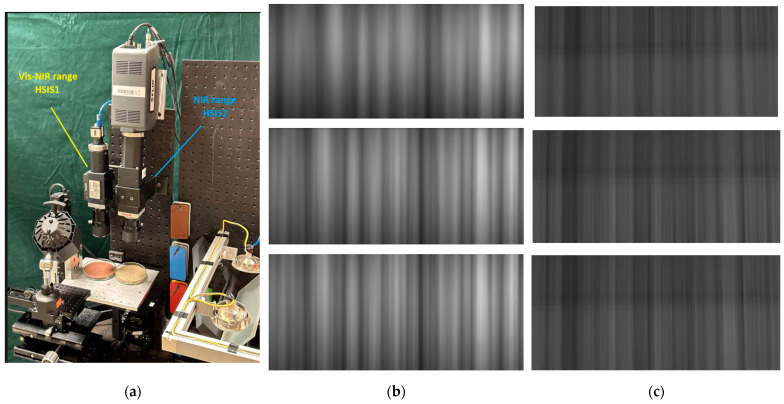
(**a**) The view of the vis-NIR (left) and NIR (right) hyperspectral imaging systems (HSISs); (**b**) examples of vis-NIR soil hyperspectral images recorded with HSIS1: at 50% (top), 90% (middle), and in-focus (bottom) positions of the focus curve; (**c**) examples of NIR soil hyperspectral images recorded with HSIS2: at 50% (top), 90% (middle), and in-focus (bottom) positions of the focus curve.

The remaining two setups, referred to as HSIS1 and HSIS2 in this paper, incorporate a single objective lens positioned in front of the spectrometer. Specifically, a 55 mm focal length partially telecentric imaging lens (1.0×–0.4×, FL 55 mm, from Edmund Optics) is utilized (Figure 2a). These setups employ a 45-degree incident and 90-degree detection (d90R45) configuration for illumination [8]. Two quartz–halogen lamps (150 W each) are arranged on the left and right sides of the objective lens, providing uniform irradiation over a sample surface area of approximately 50 mm^2^, which significantly exceeds the FOV of the corresponding HSIS. These HSIS setups are primarily employed in studies on soil moisture analyses.

### 2.6. Instrument Calibration

To spectrally calibrate and determine the spectral resolution of the HSIS, several types of fluorescence calibration lamps (particularly neon, mercury, and xenon lamps from Pen-Ray, Spectral Calibration Lamps) with reference spectral lines listed in the NIST Atomic Spectra Database were employed. Additionally, a series of lasers with known wavelengths at 0.47, 0.53, 1.15, 1.35, and 1.55 μm were utilized. The beams from these lasers were diffusely scattered inside an integrating sphere and subsequently used for calibration purposes. The spectral resolutions of each system are depicted in Table 1.

The alignment of the sensor array and corresponding spectrometers was conducted using the standard USAF 1951 test chart, following the procedures described in [48]. Alignment validation was performed utilizing a calibrated gauge—a standard glass ruler from Mitutoya (Japan) that has 100 μm separations between adjacent grid marks (this gauge was also used for the determination of the spatial resolution of the setups)—and a white paper with different thin parallel lines printed 1–3 mm apart. In order to minimize the contribution of the optical distortion of the objective lenses utilized in the studies, in alignment validation tests, we used a telecentric lens (0.5× Gold Series, F/25, Edmund Optics) for the visible- and NIR-range HSIMs.

### 2.7. Samples and Sample Preparation

To ensure robust comparisons and to draw general conclusions for selecting a particular FM, it is essential to choose a statistically sufficient number of samples that convey a variety of information and exhibit a reasonable variation in attribute features. In order to meet this requirement, different samples were chosen for the vis-NIR- and NIR-range experiments. Specifically, in the visible range, nine different samples of five types were used (Figure 1c): (i) diffuse surface (pieces of paper, both white and colored), (ii) specularly and diffusely mixed reflectance surface (colored plastic surfaces), (iii) specularly weighted reflectance surface (an aluminum plate), (iv) heterogeneous sample (a mix of ground coffee and pepper powders), and (v) wood. In the case of NIR experiments, the following samples were used: (i) wood, (ii) external and internal skin surfaces of banana, (iii) slices of apple (freshly cut and moisture wiped by paper towels), (iv) slice of cheese, (v) textile doused in biodiesel, (vi) coffee powder, and (vii) fresh bay leaves. The leaf, cheese, and fruit slice samples were placed on a stainless steel background and covered with a quartz glass plate of a 0.8 mm thickness. This was carried out to prevent the evaporation of moisture due to lamp illumination.

In addition to the aforementioned items, the FMs were examined using bare soil samples. In total, 11 different types of soil, with varying grain sizes and clay and sand content, were selected for initial experiments. Certain amounts of samples were sieved with particular sizes of 0.5 mm and 2 mm. These samples were placed into a Petri dish with a thickness of about 1 cm. These dried and sieved samples were used in current experiments. Additionally, special attention was paid to prepare an isoplanatic surface on all samples. All samples were prepared at room temperature, 23 ± 0.5 °C.

### 2.8. Image Acquisition

Hyperspectral images were acquired in the diffuse reflectance mode. A series of hyperspectral image stacks was captured for each experimental sample. Motorized movements in the Z-direction of the systems were controlled using homemade software that allowed the synchronization of the image acquisition process with lateral scanning of the distance between the imager and object plane. Images within each series of image stacks correspond to the same FOV and optical magnifications captured at finely spaced intervals along the optical axis of the corresponding hyperspectral imaging system. For each zoom value of the objective lens used, the corresponding depth of field (DOF, ∆Z) was calculated according to the expression $∆Z=\lambda /\left[4\cdot n\left(1-\sqrt{\left(1-{\left(NA/n\right)}^{2}\right)}\right)\right]$, where λ is the effective wavelength of illumination, *n* is the index of the refraction of air, and *NA* is the numerical aperture of the objective lens at a given optical magnification. Then, corresponding lateral scanning step sizes were calculated, taking into account the Nyquist criterion. In all experiments, image stacks containing from 150 to 250 images (depending on the optical zoom value) for HSIMs, and 80 to 100 images for HSISs, were captured and saved in a raw format for further processing. Each image stack is also coupled with a corresponding dark image that is captured before the series acquisition.

## 3. Results

### 3.1. Validation Phase: Video Images

**Figure 3:**
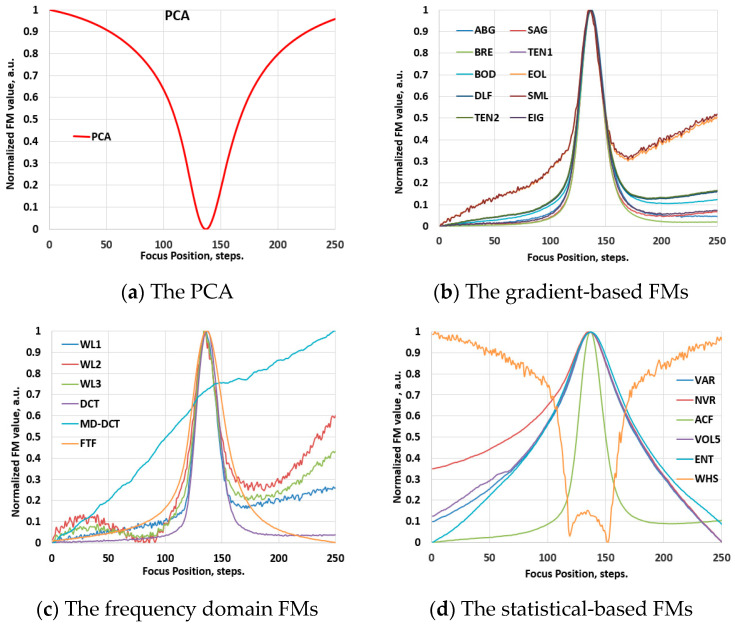
Validation phase: the normalized values of the FMs of an image stack, recorded with the visible-range video system (with the removed spectrograph from the HSIM1) at 100× optical magnification. For the abbreviations of FMs, please refer to Section 2.2.

**Figure 4:**
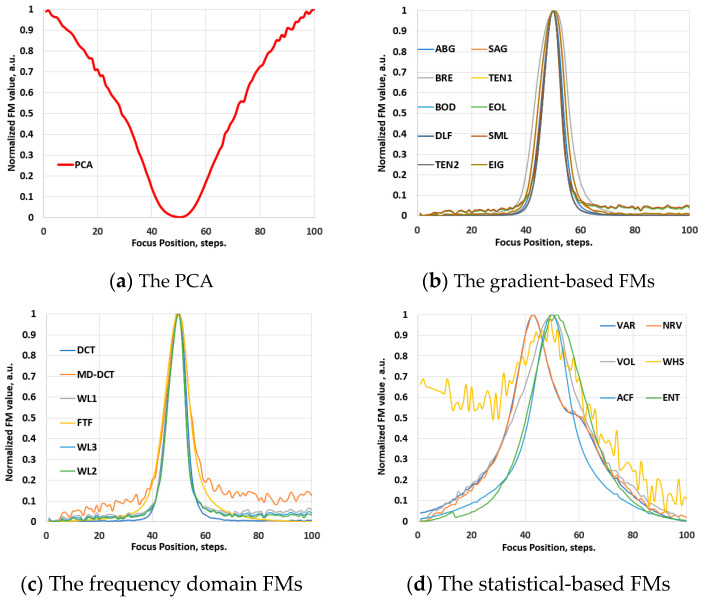
Validation phase: the normalized values of the FMs of an image stack, recorded with the NIR-range video system (with the removed spectrograph from the HSIS2) at 1× optical magnification.

Prior to examination within the scope of the hyperspectral imaging domain, the performance of the FM functions was assessed using conventional video image stacks. To preserve the optical configurations maximally, the spectrographs were removed from the Vis-NIR and NIR hyperspectral systems, transforming them into conventional video imagers. Various samples were used to record through-focus video image stacks at different optical magnifications. PCA was applied to determine the best focus position in these stacks. Figure 3a presents the result of this evaluation, illustrating the global minimum of the PCA, which was used as the reference for evaluating the performance of the FMs. Subsequently, the FMs were applied to determine the focus position in these stacks. Figure 3b–d show examples of these evaluations, illustrating typical focus curves of the FMs, which are normalized by their maximum values and categorized by families. Likewise, Figure 4a–d depict these results for the NIR range, obtained employing the same soil sample. The results indicate that the functions exhibit a reliable focus curve with a distinct extreme value and monotonic behavior, where different FM values correspond to different levels of defocusing. This behavior aligns well with state-of-the-art results [19,20,21,22]. 

Moreover, the best focus position in these video stacks was determined by proficient microscope operators and compared with that determined by PCA evaluations. An exact coincidence of the focus positions obtained using PCA and determined by an operator was observed for all video stacks. Based on these results, the focus position determined by PCA was used as the reference for the accuracy estimation of the FM in the hyperspectral imaging domain.

### 3.2. The Behavior of the FMs in Hyperspectral Images

In the evaluations, a dataset comprising 92 hyperspectral image stacks was utilized for performance assessments. This dataset encompasses a total of 72 hyperspectral image stacks of soil samples, with 36 image stacks equally distributed across both the Vis-NIR and NIR bands. Additionally, the dataset incorporates 14 image stacks within the Vis-NIR range and another 16 within the NIR range, corresponding to the objects specified in Section 2.7.

#### 3.2.1. The Vis-NIR Range

**Figure 5:**
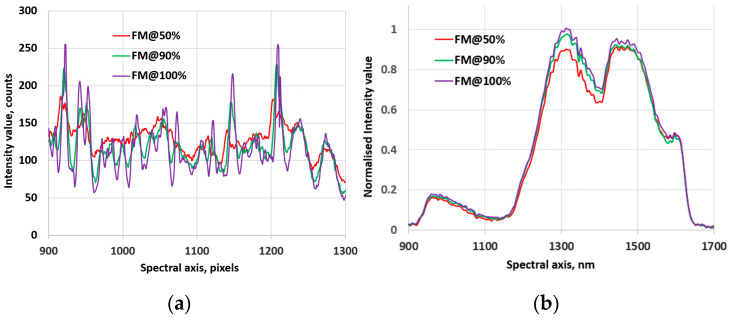
An example of an image profile along the spatial axis (**a**) and the spectral axis (**b**), where the violet curves correspond to well-focused images, while the red and green curves represent defocused images with focus measures at 50% and 90% of the maximum, respectively.

**Figure 6:**
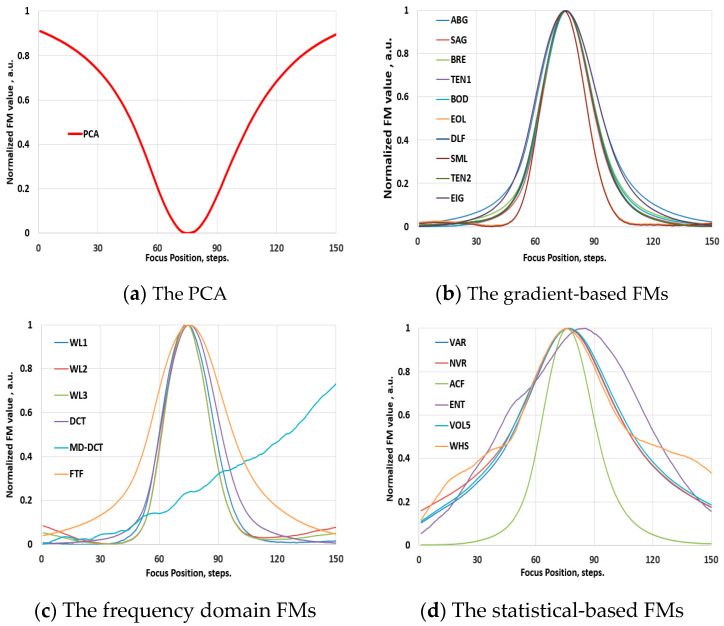
The normalized values of the FMs of an image stack, recorded with the vis-NIR-range hyperspectral system HSIS1 at 0.5× optical magnification.

Figure 2b shows an example of Vis-NIR hyperspectral images of the same soil recorded at the well-focused and defocused (corresponding to 50% (top) and 90% (middle) positions of the focus curve) focal positions. Figure 5a illustrates an example of an image profile along the spatial axis for a well-focused image and defocused images with focus measures at 50% and 90% of the maximum, respectively. Figure 6a–d display a series of focus curves for each family of FM evaluated for the same stack using raw Vis-NIR hyperspectral images (HSIS1 at 0.5× optical magnification). Practically similar behaviors of focus curves were observed for all hyperspectral image stacks in the dataset recorded using the soil samples and HSIS1. 

**Figure 7:**
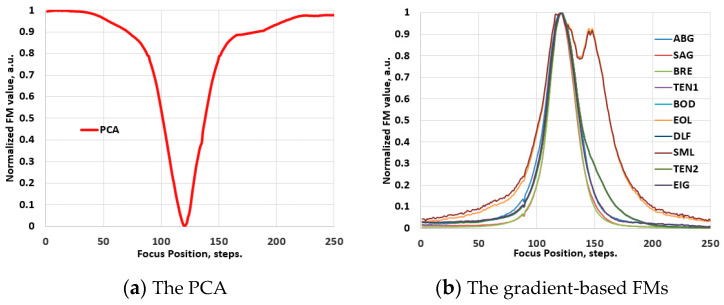

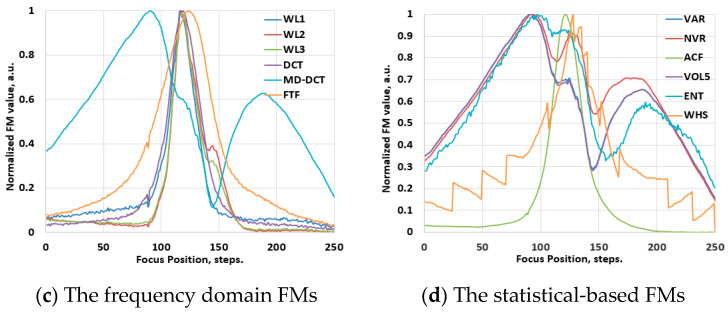
The normalized values of the FMs of an image stack, recorded with the vis-NIR-range hyperspectral system HSIM1 at 100× optical magnification.

However, in experiments with the HSIM1, an increase in optical magnification resulted in the deterioration of focus curve behavior in terms of the width and noise level, as it is shown in Figure 7a–d (HSIM1 at 100× optical magnification). Nevertheless, the position of the best focus remained mostly unchanged, which was most prominent for gradient-based and frequency-based FMs. Except for the SAG and EOL, due to their side lobes, all gradient-based FMs demonstrated behavior close to ideal curves. In some cases, with increased optical magnification, a second false peak appeared for frequency-based FMs. However, the width of the focus curve, especially for wavelet-based FMs, reacted only slightly to the optical magnification. Except for the ACF, all FMs in the statistics-based FM demonstrated degradation with the increase in the optical magnification of the imaging system. It is worth noting that in the experiments with the HSIS1, the behavior of focus curves was more consistent with the variation in the optical magnification from 0.5× to 1×.

#### 3.2.2. The NIR Range

**Figure 8:**
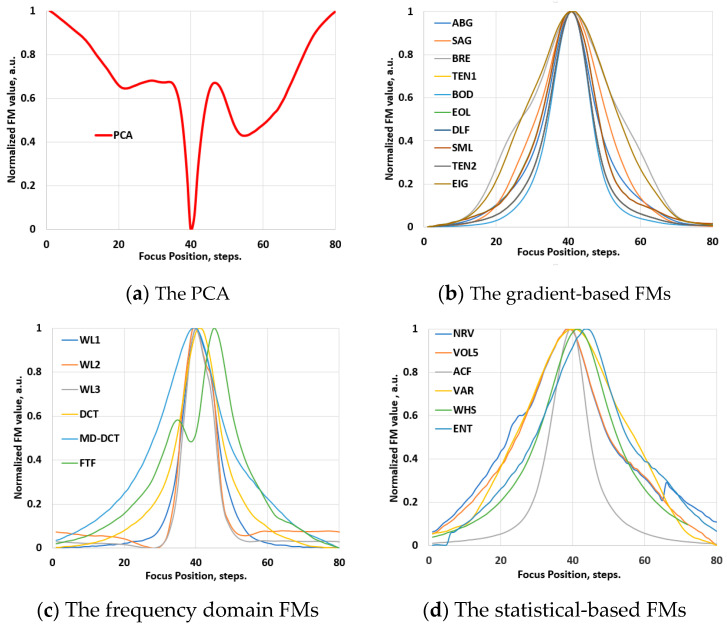
The normalized values of the FMs of an image stack, recorded with the NIR-range hyperspectral system HSIM2 at 25× optical magnification.

Finally, we assessed the performance of the FMs on the NIR-range hyperspectral im-age stacks. Figure 2c shows an example of NIR hyperspectral images (obtained using the HSIS2 and a soil sample) recorded at the well-focused (the image in the bottom of Figure 2c) and defocused (corresponding to 50% (top) and 90% (middle) positions of the focus curve) focal positions. Figure 5b illustrates an example of an image profile along the spectral axis for a well-focused image and defocused images with focus measures at 50% and 90% of the maximum, respectively. A series of focus curves of each family of FM evaluated for the same stack using raw NIR hyperspectral images are shown in Figure 8a–d. Analogous to the validation and the Vis-NIR case, here, due to the lack of space, we solely present the results of one hyperspectral image stack, corresponding to the same soil sample. In contrast to the results observed in the video mode datasets and hyperspectral images in the vis-NIR range, where practically all FMs exhibited a characteristic behavior consistent with focus curves featuring a global extremum, several FMs (the Mid-DCT and most statistics-based FMs, except for the ACF) in the NIR-range hyperspectral image series displayed no extremum whatsoever. Similarly, the effectiveness of PCA also fluctuated in these stacks. Especially at high magnifications (around 50×) of the HISM2, the fluctuations became more prominent. While the shape of the PCA curve improves with an increase in the number of involved components, the general noisy behavior of the focus curves of most FMs and PCA increases the overall evaluation uncertainty. To address this issue, we observed that averaging images up to 10 at each focal position did not notably enhance the quality of the FMs. Subsequently, we implemented a sub-sampling (i.e., a selection of a region of interest, i.e., ROI) option for each individual image within the stack before evaluating the FMs. This approach significantly enhanced the performance of the FMs and PCA focus curves. Therefore, in evaluations involving noisy NIR images, the assessments were conducted by applying ROI to the images, as it is described in the next subsection.

### 3.3. Robustness of the FMs

In this study, the speed of the autofocusing process will not be examined, as will be discussed later. However, extensive investigation was conducted to understand statistically the impact of sub-sampling (i.e., ROI) of the images in a stack, which enhances the speed of the autofocusing process. While the acceleration of FM evaluations through ROI selection is evident due to the reduction in the number of pixels in proportion to the ROI area compared to the entire image area, the extent to which the performance of FMs will be affected by sub-scaling remains unclear. Various windows sizes and locations of ROI selection were applied to image stacks prior to the assessment of FM performance.

**Figure 9:**
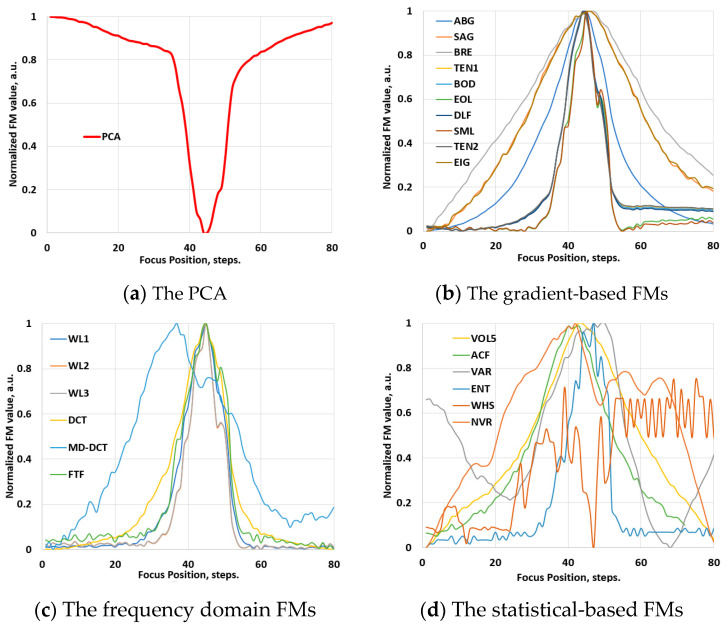
Noisy Hyperspectral Images. The normalized values of the FMs of an image stack, recorded with the NIR-range hyperspectral system HSIS2 at 0.5× optical magnification.

Finally, we assessed the robustness of the FMs against artificially generated noise and nonhomogeneous illumination. For the first purpose, we added Poisson noise (using the Matlab function “poissrnd” with parameters of 10, 25, 50, and 100) to each original image in an image stack obtained in the visible range and conducted focus evaluations. For the second purpose, a luminance gradient, represented as a quadratic polynomial function with maximum intensity values of 0.8, 0.9, and 1, and a minimum value of 0, was applied as a gray-level image, multiplied with the original images [23]. Figure 9a–d demonstrate an example of the focus curve behavior in noisy image stacks. Through the analysis of more than 20 hyperspectral image stacks, ABG, WL3, and ACF were found to be more stable in focusing on noisy images. The results of these simulations were utilized in subsequent ranking evaluations of the FMs, as will be described in the next section.

## 4. Discussion

### 4.1. Results of Ranking Evaluations

The calculation of performance indices is carried out using normalized focus curves. For each normalized focus curve, the distance parameter, denoted as *D*, is determined by calculating the difference between the observed $\left[{d_{acc\;},\;d}_{uni\;},\;{d_{50\;},\;d}_{90\;},d_{smo\;}\right]\;$ value and the ideal value $\left[0,\;0,\;0,\;0,\;0\right]\;$ for the corresponding criteria. A smaller distance value suggests better performance of that FM when ranked based on that parameter. To establish an overall ranking, the Euclidean length of each focus curve is calculated using the following formula:

$$D=\sqrt{d_{acc}^{2}+d_{uni}^{2}+d_{50}^{2}+d_{90}^{2}+d_{smo}^{2}}$$

where each distance is normalized to its maximum value. This ensures that in the overall scoring, each distance carries equal weight. Consequently, the best FM will have the lowest overall score.

#### 4.1.1. Ranking Evaluations for the Conventional Images

First, we conducted an evaluation of conventional monochrome video image stacks. Table 2 summarizes the ranking of the FMs based on individual criterion distances and overall scores, calculated from the average of 40 image stacks of samples as described in Section 3.3 and soil samples. TEN1 and BOD were identified as providing nearly identical top-ranking performance among the gradient-based FMs. While BRE and ABG FMs exhibited the highest accuracy and behavior similar to TEN1, BOD, and TEN2 in terms of curve width, a false maximum was observed for these FMs, particularly at the highest magnification. However, at low magnifications ranging from 0.5× to 1×, no false maximum was observed, and they demonstrated overall performance comparable to the leading FMs. ***WL3*** exhibited the best overall performance among all evaluated FMs. Unlike other frequency-based FMs, no false maximum was observed for ***WL3*** at higher magnifications. Among the statistics-based FMs, ***NVR*** was identified as the superior algorithm. Similarly, Table 3 presents the ranking of the FMs based on conventional video images (a dataset containing 35 image stacks) captured with NIR imaging systems. Among their respective FM families, ***TEN1***, ***WL3***, and ***VOL5*** emerged as the top-performing algorithms, with ***TEN1*** demonstrating the best performance among all FMs.

#### 4.1.2. Ranking Evaluations for the Hyperspectral Images

Table 4 summarizes the individual performance and ranks within each respective family of all evaluated FMs in the context of the vis-NIR hyperspectral domain. As seen from Table 4, in this domain, the first six gradient-based FMs demonstrate very close performance to each other, with the ***BRE*** function resulting in stable performance across all investigated samples, including noisy images. This is followed by the best performance of ***WL3*** and ***ACF*** algorithms in the frequency and statistical groups, respectively. Generally, the three wavelet-based FMs demonstrate good performance in terms of accuracy and curve width, except for unimodality. Particularly, with the increase in optical magnification, a side lobe appears around the main peak of ***WL1*** and ***WL2*** focus curves.

While statistics-based algorithms are recognized for their strong performance with conventional images [20,22,28,32], our experiments have shown that they underperform with hyperspectral images. For the vis-NIR domain images, ***AFC*** was found to provide the best overall performance, comparable to the leaders of the other two families of FMs. Although the accuracy of the ***ENT*** and ***VOL5*** functions was similar to that of ***AFC***, they underperformed in other criteria.

Finally, Table 5 summarizes the individual performance and ranks within each respective family of all evaluated FMs in the context of the NIR hyperspectral domain. By comparing with Table 4, it can be seen that in the gradient- and statistical-based groups, the ranking of FMs was changed, with no change in the frequency-domain FMs. This shows that these algorithms, especially wavelet-based ones, are the most robust to the sub-sampling and noisy images. BOD and ABG demonstrated high performance in locating the best focus position very near to that determined by PCA; however, the width of the ABG focus curve deteriorates with noise in the image. Therefore, BOD was found to demonstrate the best performance in the NIR spectral band hyperspectral images. This is followed by WL3 in the frequency domain and NVR in the statistical-based FM family. In the latter group, ENT demonstrates very similar overall performance to NVR.

## 5. Conclusions

Autofocusing is one of the fundamental techniques influencing the efficiency of automated digital imaging systems. An image-based autofocusing method, based on image sharpness information (i.e., FM) to determine the best focus position, predominates in the field. Since FMs are domain-specific, their performance is evaluated using verified focus positions. In this study, we employed PCA to define the optimal focus position in hyperspectral imaging stacks, where images lack clear visual information. Then, we conducted a systematic analysis of the performance of twenty widely utilized FMs for autofocusing in hyperspectral imaging systems. We acquired a diverse number of hyperspectral image stacks, encompassing high (25×–100×) and low (0.1×–0.5×) optical magnifications, utilizing a variety of four pushbroom imaging spectrometer-based systems operating within the Visible–NIR (400–1000 nm) and NIR (900–1700 nm) spectral ranges. The performance of the various focus measures was assessed through experiments conducted under diverse conditions, including variations in the optical magnification, illumination, spectral band, image noise level, nonuniform illumination, and window size.

Furthermore, a ranking methodology, inherent to hyperspectral imaging systems utilized in remote sensing, was proposed. Based on these criteria, the overall performance of the focus measures was evaluated both in a group and individually. Experimental results demonstrate that gradient-based focus measures are the most rapid and reliable operators in the field, while wavelet-based algorithms are the most robust to sub-sampling and noise in hyperspectral images.

## Figures and Tables

**Table 1:** Summary of HSIS.

Setup	HISM1	HISM2	HSIS1	HSIS2
Spectral range	400–1000 nm	900–1700 nm	400–1000 nm	900–1700 nm
Entrance slit, width × height	30 μm × 9.8 mm	50 μm × 9.8 mm	25 μm × 9.8 mm	25 μm × 9.8 mm
Spectral resolution	3.8 nm/pixel	6.7 nm/pixel	1.3 nm/pixel	4.1 nm/pixel
Imaging array	Pixel Fly, PCO	XenIcs, XEVA-17, InGaAs	EO, DCC3240x	FLIR, A6261, InGaAs
Array resolution, pixels	1024 × 1392	256 × 320	1280 × 1024	640 × 512
Dynamic range	14 bits	12 bits	12 bits	14 bits
Magnification	50× to 100×	25× to 50×	0.1× to 0.5×	0.1× to 0.5×

**Table 2:** Validation phase. Ranking of FMs for vis-NIR conventional video images.

FM	Accuracy	Unimodality	Width at 50%	Width at 90%	Smoothness	Overall Score	Ranking
TEN1	0.10	0.00	0.59	0.37	0.59	0.92	1
BOD	0.15	0.00	0.54	0.46	0.58	0.93	2
TEN2	0.10	0.00	0.56	0.41	0.62	0.94	3
BRE	0.05	0.05	0.59	0.47	0.60	0.97	4
ABG	0.05	0.05	0.68	0.60	0.59	1.08	5
SAG	0.11	0.11	0.59	0.53	0.81	1.14	6
EIG	0.32	0.12	0.62	0.58	0.77	1.20	7
SML	0.59	0.33	0.78	0.81	0.87	1.57	8
EOL	0.20	1.00	0.67	0.70	1.00	1.73	9
DLF	1.00	0.45	1.00	1.00	0.75	1.94	10
WL3	0.10	0.00	0.55	0.39	0.49	0.84	1
WL2	0.21	0.19	0.57	0.47	0.51	0.94	2
WL1	0.18	0.11	0.59	0.48	0.53	0.95	3
FTF	0.83	0.92	0.69	0.76	0.67	1.74	4
DCT	1.00	1.00	0.74	0.90	0.65	1.94	5
MD-DCT	0.79	0.71	1.00	1.00	1.00	2.03	6
NVR	0.41	0.45	0.43	0.31	0.71	1.08	1
ENT	0.31	0.40	0.50	0.48	0.75	1.14	2
VOL5	0.40	0.40	0.61	0.59	0.85	1.33	3
ACF	0.51	0.55	0.69	0.55	0.71	1.36	4
VAR	0.46	0.60	0.85	0.77	0.78	1.58	5
WHS	1.00	1.00	1.00	1.00	0.92	2.20	6

**Table 3:** Validation phase. Ranking of FMs for NIR conventional video images.

FM	Accuracy	Unimodality	Width at 50%	Width at 90%	Smoothness	Overall Score	Ranking
TEN1	0.30	0.02	0.55	0.45	0.50	0.92	1
TEN2	0.21	0.06	0.61	0.59	0.45	0.99	2
ABG	0.23	0.02	0.78	0.56	0.49	1.10	3
BRE	0.58	0.07	0.59	0.52	0.59	1.14	4
BOD	0.51	0.04	0.67	0.61	0.51	1.16	5
SAG	0.62	0.14	0.69	0.73	0.61	1.34	6
EIG	0.42	0.32	0.82	0.88	0.67	1.47	7
EOL	0.73	0.32	0.96	0.78	1.00	1.78	8
DLF	1.00	0.91	0.87	0.93	0.85	2.04	9
SML	0.63	1.00	1.00	1.00	0.91	2.06	10
WL3	0.33	0.12	0.50	0.48	0.59	0.98	1
WL2	0.37	0.22	0.57	0.51	0.45	0.99	2
WL1	0.41	0.32	0.75	0.65	0.59	1.27	3
FTF	0.63	0.32	0.96	0.87	0.67	1.62	4
DCT	0.85	1.00	0.87	0.93	0.95	2.06	5
MD-DCT	1.00	0.91	1.00	1.00	1.00	2.20	6
VOL5	0.40	0.21	0.61	0.49	0.65	1.11	1
ACF	0.41	0.09	0.59	0.65	0.60	1.14	2
ENT	0.31	0.42	0.56	0.60	0.75	1.23	3
NVR	0.48	0.13	0.63	0.71	0.71	1.29	4
VAR	0.76	1.00	0.85	0.77	1.00	1.97	5
WHS	1.00	0.96	1.00	1.00	0.91	2.18	6

**Table 4:** The ranking of the FMs for the vis-NIR spectral band hyperspectral images.

FM	Accuracy	Unimodality	Width at 50%	Width at 90%	Smoothness	Overall Score	Ranking
BRE	0.19	0.07	0.39	0.35	0.30	0.64	1
BOD	0.21	0.10	0.37	0.35	0.35	0.66	2
EIG	0.30	0.12	0.32	0.38	0.32	0.67	3
SAG	0.29	0.14	0.29	0.33	0.41	0.68	4
TEN1	0.23	0.08	0.45	0.36	0.31	0.70	5
TEN2	0.21	0.09	0.41	0.32	0.42	0.71	6
ABG	0.29	0.11	0.37	0.40	0.49	0.80	7
DLF	0.67	0.45	0.87	0.93	0.55	1.60	8
EOL	1.00	1.00	1.00	0.88	1.00	2.19	9
SML	0.93	1.00	1.00	1.00	0.97	2.19	10
WL3	0.23	0.08	0.38	0.35	0.31	0.65	1
WL2	0.24	0.12	0.39	0.41	0.31	0.70	2
WL1	0.27	0.12	0.41	0.43	0.34	0.75	3
FTF	0.53	1.00	1.00	0.87	0.67	1.87	4
DCT	0.75	0.90	0.81	1.00	0.95	1.98	5
MD-DCT	1.00	0.91	0.93	1.00	1.00	2.17	6
ACF	0.41	0.20	0.33	0.34	0.25	0.70	1
VOL5	0.45	0.31	0.61	0.59	0.36	1.07	2
ENT	0.41	0.32	0.68	0.56	0.45	1.12	3
VAR	1.00	0.43	1.00	0.67	0.71	1.77	4
NVR	0.94	0.37	0.91	1.00	0.79	1.86	5
WHS	0.80	1.00	0.83	0.81	1.00	2.00	6

**Table 5:** The ranking of the FMs for the NIR spectral band hyperspectral images.

FM	Accuracy	Unimodality	Width at 50%	Width at 90%	Smoothness	Overall Score	Ranking
BOD	0.05	0.04	0.37	0.35	0.51	0.72	1
TEN1	0.15	0.02	0.60	0.52	0.50	0.95	2
TEN2	0.25	0.02	0.64	0.58	0.45	1.01	3
ABG	0.10	0.02	0.70	0.62	0.49	1.06	4
BRE	0.19	0.07	0.61	0.68	0.59	1.11	5
SAG	0.52	0.24	0.59	0.60	0.61	1.19	6
EIG	0.32	0.38	0.42	0.43	1.00	1.27	7
SML	1.00	0.80	0.50	0.55	0.71	1.64	8
DLF	0.64	0.71	0.66	0.93	0.86	1.72	9
EOL	0.73	1.00	1.00	1.00	0.85	2.06	10
WL3	0.13	0.12	0.50	0.47	0.40	0.81	1
WL2	0.16	0.12	0.59	0.56	0.45	0.95	2
WL1	0.31	0.32	0.62	0.63	0.57	1.14	3
FTF	1.00	1.00	0.86	0.87	1.00	2.12	4
DCT	0.85	0.90	0.67	0.73	0.70	1.73	5
MD-DCT	1.00	0.80	1.00	1.00	0.88	2.10	6
NVR	0.44	0.23	0.59	0.57	0.41	1.04	1
ENT	0.49	0.12	0.61	0.60	0.35	1.05	2
ACF	0.57	0.31	0.60	0.55	0.68	1.24	3
VOL5	0.65	0.29	0.71	0.60	0.63	1.33	4
VAR	1.00	1.00	0.85	0.86	0.83	2.04	5
WHS	0.91	0.80	1.00	1.00	1.00	2.11	6

## Data Availability

The data presented in this study are available on request from the corresponding author.
